# Comment on “UV Absorption Spectroscopy of the Conformer-Dependent Reactivity of the Four Carbon Criegee Intermediate of Methyl Vinyl Ketone Oxide: An Ab initio Quantum Dynamics Study”

**DOI:** 10.1021/acs.jpca.4c00105

**Published:** 2024-01-31

**Authors:** Yen-Hsiu Lin, Kaito Takahashi, Jim Jr-Min Lin

**Affiliations:** † Institute of Atomic and Molecular Sciences, Academia Sinica, Taipei 106319, Taiwan; ‡ Department of Chemistry, National Taiwan University, Taipei 106319, Taiwan

In the article “UV Absorption Spectroscopy of the Conformer-Dependent Reactivity of the Four Carbon Criegee Intermediate of Methyl Vinyl Ketone Oxide: An Ab initio Quantum Dynamics Study”,^1^ Nikoobakht presented an extended theoretical analysis of the photodissociation dynamics of the four-carbon Criegee intermediate CH_2_=CH(CH_3_)COO or methyl vinyl ketone oxide (MVKO). Sophisticated theoretical tools, including multireference electronic wave functions combined with a wavepacket propagation treatment for the two coupled electronic states, were used to simulate the UV absorption spectra of *syn*- and *anti*-conformers of MVKO. The resulting theoretical spectra were also compared with the reported experimental spectra by Caravan et al.,^2^ Vansco et al.,^3^ and Lin et al.^4^

We have no issue with the calculation results; however, in Figure 7 of that article,^1^ the experimental data of Lin et al. [Figure 3]^4^ were misinterpreted as the spectrum coming from MVKO. The “experimental” spectrum (blue line of Figure 7 by Nikoobakht)^1^ is actually a raw experimental spectrum that includes the contributions of MVKO, IO (iodine monoxide)^5^ and I_2_ (iodine molecule).^6^ The spectral shapes of these three species are distinctively different; thus, we determined their contributions and plotted them in that Figure 3 with various colors (total 10 colored lines were used to show everything).^4^ In particular, the sharp spectral peaks at 412, 419, 427, 436, 445, and 455 nm belong to the absorption of IO,^5^ not of MVKO, as mentioned in the figure caption “... In addition to the MVKO absorption band peaking at 371 nm, the signals of byproducts IO (sharp peaks between 400 and 460 nm) and I_2_ (broad absorption from 430 to 520 nm) are also observed.”^4^

In fact, Lin et al.^4^ had no intention to report the spectral shape of the MVKO electronic spectrum but to report the absolute absorption cross section of MVKO at 351 nm. The spectral shape of MVKO used in the work of Lin et al.^4^ is essentially the same as that of Caravan et al.,^2^ which is broad and structureless, as shown in Figure 11 of the same publication.^4^

Therefore, the sentences in the Introduction by Nikoobakht^1^ (the first paragraph of page 10092, copied below):

“In the recent work of Lin *et al*., the authors used the laser-depletion method and measured the absorption spectrum (corresponding to the B^1^A′ ← X^1^A′ electronic transition) with a high resolution in the wavelength range 360–480 nm.^8^ They observed well-resolved peak structures on the low-energy side of the spectrum. In contrast to the recent measurement carried out by Lin *et al.*, the two reported spectra in refs 3 and 4 do not show any sizable structures at the low energy side of the spectrum.“

are a misinterpretation of the experimental results of Lin et al.^4^

Since the “well-resolved peak structure” (oscillation) at ca. 400–460 nm belongs to IO, it has no relevance to the theoretical spectra of MVKO. Here, we are not arguing whether MVKO has an oscillatory structure in that spectral region. Rather, we comment that connecting such an oscillation in the theoretical spectra to the oscillatory structure seen in the “raw” experimental spectra is a misinterpretation. Thus, the sentence in the abstract^1^

“... which can explain the oscillatory structures appearing in the long wavelength range of 360–480 nm of the spectrum.”

may not be applicable.
